# Recent Developments in Semiconductor-Based Photocatalytic Degradation of Antiviral Drug Pollutants

**DOI:** 10.3390/toxics11080692

**Published:** 2023-08-11

**Authors:** Zhaocheng Zhang, Dongyang He, Siyu Zhao, Jiao Qu

**Affiliations:** 1 Key Laboratory of Geographical Processes and Ecological Security of Changbai Mountains, Ministry of Education, School of Geographical Sciences, Northeast Normal University, Changchun 130024, China; zhangzc363@nenu.edu.cn; 2 School of Environment, Northeast Normal University, Changchun 130117, China; syzhao999@nenu.edu.cn

**Keywords:** antiviral drug, photocatalysis, semiconductor, photocatalytic mechanism

## Abstract

The prevalence of antiviral drugs (ATVs) has seen a substantial increase in response to the COVID-19 pandemic, leading to heightened concentrations of these pharmaceuticals in wastewater systems. The hydrophilic nature of ATVs has been identified as a significant factor contributing to the low degradation efficiency observed in wastewater treatment plants. This characteristic often necessitates the implementation of additional treatment steps to achieve the complete degradation of ATVs. Semiconductor-based photocatalysis has garnered considerable attention due to its promising potential in achieving efficient degradation rates and subsequent mineralization of pollutants, leveraging the inexhaustible energy of sunlight. However, in recent years, there have been few comprehensive reports that have thoroughly summarized and analyzed the application of photocatalysis for the removal of ATVs. This review commences by summarizing the types and occurrence of ATVs. Furthermore, it places a significant emphasis on delivering a comprehensive summary and analysis of the characteristics pertaining to the photocatalytic elimination of ATVs, utilizing semiconductor photocatalysts such as metal oxides, doped metal oxides, and heterojunctions. Ultimately, the review sheds light on the identified research gaps and key concerns, offering invaluable insights to steer future investigations in this field.

## 1. Introduction

Antiviral drugs (ATVs) are a distinct category of therapeutic agents utilized to treat various viral infections, encompassing both specific and broad-spectrum activity [1]. In recent years, there has been a persistent global rise in the occurrence of viral infections, resulting in epidemic and pandemic outbreaks. The outbreaks of influenza and recent global pandemics, such as severe acute respiratory syndrome coronavirus 2 (SARS-CoV-2), have underscored the substantial impact of viral infections as a leading cause of mortality worldwide [2,3,4]. The development of ATVs has been a critical endeavor in the realm of scientific research, driven by the pressing global challenge of viral infections. By dedicating focused efforts to disease control and mitigation, these pharmaceutical interventions possess immense potential in curbing the morbidity and mortality associated with viral outbreaks. In 1963, the United States Food and Drug Administration (FDA) recognized idoxuridine as the first antiviral compound for the treatment of herpes simplex virus (HSV) keratitis [5,6]. Subsequently, a diverse range of ATVs emerged as therapeutic interventions for the treatment of various viral infections, encompassing influenza, herpes simplex virus (HSVs), hepatitis, human immunodeficiency virus (HIV), and coxsackievirus [7,8,9]. Nevertheless, the extensive use of ATVs in medical settings and their discharge into wastewater systems have led to growing concern regarding their potential as emerging anthropogenic pollutants capable of entering water environments [10]. The presence of diverse ATVs in aqueous environments has sparked growing apprehension regarding their potential adverse effects on human health [11,12,13,14,15,16]. It has been observed that these compounds exhibit limited degradation efficiency in wastewater treatment plants (WWTPs) due to their hydrophilic nature [17]. Consequently, the contamination of aquatic systems with ATVs through effluents from WWTPs is a plausible occurrence [18]. Thus, there is an urgent need to develop efficient methods for the treatment of ATV-contaminated waters, aiming to reduce or completely eliminate pollutants.

Among various methods, advanced oxidation processes (AOPs), such as ozone-based, Fenton/Fenton-like, electrochemical, and photocatalytic processes, have shown promise for the efficient elimination of ATVs [19,20,21,22]. AOPs are known for their ability to efficiently remove persistent and toxic contaminants that are challenging to eliminate using conventional treatment methods. These approaches are based on the in situ generation of a potent oxidizing agent, such as hydroxyl radicals (•OH), at a concentration sufficient to effectively decontaminate water systems. Heterogeneous photocatalysis, in particular, has been the subject of extensive research owing to its inherent advantages, such as the absence of additional chemicals, low energy consumption, straightforward equipment, mild operating condition, and cost-effectiveness [23,24,25]. Harnessing the potential of photocatalysis, a multitude of photocatalytic materials have been developed, exhibiting remarkable photocatalytic activity. This progress holds great promise for the efficient degradation of ATV-contaminated waters. Although there are numerous reviews on the photocatalytic degradation of antibiotics and pharmaceuticals, there is a limited amount of research on the photocatalytic degradation of ATVs.

This review begins by providing an overview of the types and occurrence of ATVs in aqueous environments. Subsequently, it emphasizes the provision of a comprehensive summary and analysis of the characteristics associated with the photocatalytic degradation of ATVs. Specifically, it highlights the influential role of bandgap on photocatalytic activity, explores the intricate mechanism of free radical degradation, and examines the kinetics involved in heterogeneous photocatalysis. By elucidating their interplay and implications, valuable insights are gained for the optimization of photocatalytic processes in the context of AVT degradation. Furthermore, this review comprehensively discusses the primary challenges and future directions regarding the application of photocatalysis in the practical advanced wastewater treatment of ATVs.

## 2. Types of ATVs

ATVs assume a pivotal role in curbing infectivity, ameliorating clinical manifestation, and curtailing the duration of illness. ATVs exert their effects by interrupting the intricate viral replication cycle at distinct stages [26]. Although many antiviral infections tend to resolve spontaneously due to the proficient immune system of the host, there has been a steady increase in mortality rates attributed to these pathogenic agents despite ongoing efforts to enhance our understanding of viral infections and their management [27,28,29]. Thus, the imperative for the development of ATVs therapeutics to effectively control and combat viral infections remains evident. Presently, ATVs are classified into three principal viral cohorts, encompassing herpesviruses, hepatitis viruses, and influenza viruses, alongside additional agents specifically designed for the treatment of HIV and coxsackievirus infections [30]. The notable ATVs are presented in Table 1. The ATVs are classified into 13 distinct functional groups, each exhibiting specific mechanism that effectively impede viral replication and propagation [9,31,32].

Antiretroviral drugs (ARVs) encompass a class of pharmaceutical agents specifically designed to combat retroviral infections, with a primary focus on human immunodeficiency virus type 1 (HIV-1) [33,34]. The administration of these ARVs confers substantial extensions on the lifespan of individuals afflicted with HIV, thereby yielding profound impacts on disease management. Categorized into six principal subdivisions encompassing nucleoside/nucleotide reverse transcriptase inhibitors (NRTIs), non-nucleoside reverse transcriptase inhibitors (NNRTIs), integrase inhibitors, protease inhibitors, and entry and fusion inhibitors, as well as p450-3A inhibitors, these pharmaceutical interventions exemplify the multifaceted approaches employed to combat HIV infection [35,36]. Abacavir, zidovudine, lamivudine, stavudine, and nevirapine stand out as the prevailing antiretroviral agents employed in the treatment of retroviral infections, exhibiting widespread utilization within clinical practice [37]. Their synergistic administration serves to augment the therapeutic efficacy, further enhancing the potential for achieving viral clearance in individuals afflicted with HIV.

HSVs, belonging to the herpesviridae family, represent prevalent human pathogens characterized by their enveloped, double-stranded DNA viral genome [38,39]. Predominantly manifesting in the oral and genital regions, HSVs exhibit distinct clinical presentations. In children, certain strains contribute to the development of chickenpox, potentially leading to complications such as encephalitis and pneumonia, while in adults, specific HSVs types can induce neuralgia and nerve palsy [40,41]. The HSVs encompass a spectrum of viral agents, including the highly susceptible herpes simplex virus type 1 (HSV-1), herpes simplex virus type 2 (HSV-2), varicella-zoster virus, cytomegalovirus, and Epstein-Barr virus [42]. Acyclovir, classified as an antiherpetic antiviral agent, plays a pivotal role as a therapeutic intervention for the management of herpes simplex viruses, including HSV-1, HSV-2, and varicella-zoster virus (VZV) infections [43,44]. The therapeutic efficacy of acyclovir may be compromised due to its physicochemical characteristics, characterized by low water solubility, limited membrane permeability, and modest oral bioavailability ranging between 15 and 30% [44]. Famciclovir (FCV), a prodrug designed to enhance the bioavailability of penciclovir, represents an important addition to the armamentarium of antiviral therapeutics. Both penciclovir and famciclovir exhibit potent antiherpetic activity, effectively targeting HSV-1, HSV-2, and VZV infections [45,46,47].

Influenza, a respiratory infectious disease, assumes a prominent position among the most lethal illnesses within the realm of infectious diseases, owing to its swift transmission dynamics. Influenza viruses are categorized into distinct types, namely A, B, and C, based on their matrix proteins and nucleoproteins, delineating their molecular characteristics [48,49]. While influenza can present as a relatively benign ailment in certain instances, it carries the potential for severe outcomes, including hospitalization and mortality, underscoring the variable clinical spectrum of this infectious disease [50]. The global burden of influenza epidemics is strikingly evident, with an estimated annual transmission of approximately 3 to 5 million severe infections, culminating in 290,000 to 650,000 respiratory-related fatalities worldwide [51]. While vaccination stands as a pivotal measure in mitigating influenza, its efficacy is comparatively limited in special populations, including children, the elderly, and individuals with compromised immune systems [52]. Moreover, given the time-intensive nature of vaccine production, which typically spans a minimum of six months, ATVs emerge as a valuable adjunct to complement the preventive strategies. Two distinct classes of antiviral agents, namely adamantanes (amantadine and rimantadine) and neuraminidase inhibitors (NAIs) (oseltamivir and zanamivir), have emerged as therapeutic options for the management of influenza infection [53].

In 2019, a novel coronavirus (COVID-19) associated with respiratory diseases in humans was discovered [54,55]. In March 2020, the World Health Organization (WHO) officially designated the newly identified COVID-19, also referred to as SARS-CoV-2, as a global pandemic due to its significant morbidity and mortality impact [56,57]. While no specific pharmaceutical intervention has been identified for the targeted treatment of COVID-19, clinical investigations have been conducted to evaluate the potential efficacy of several drugs, including favipiravir, remdesivir, hydroxychloroquine, azithromycin, and chloroquine [58]. Favipiravir, a potent RNA virus polymerase inhibitor, exhibits notable antiviral efficacy against a range of RNA viruses [59]. Remdesivir, an adenosine nucleotide analog, has emerged as a therapeutic agent for the management of COVID-19 in the United States, specifically indicated for individuals aged 12 years and above [60].

## 3. Occurrence of ATVs in Aqueous Environments

ATVs have been detected in various aquatic environments, including untreated wastewater, effluents from wastewater treatment plants, surface water, and groundwater. The release of ATVs into the environment can occur through three principal pathways: discharge from pharmaceutical industry effluents, the improper disposal of medical waste, and the discarding of expired, unused, or unwanted medications [61]. In particular, the elimination of ATVs within WWTPs is often incomplete, leading to their potential dissemination throughout the environment via a hierarchical cascade of pathways.

Given the escalating usage of ATVs, their release into the environment has become an unavoidable consequence. The significant removal of acyclovir, lamivudine, and abacavir was observed in WWTPs, indicating their efficient elimination during the treatment process. Conversely, nevirapine, zidovudine, and oseltamivir were detected in comparable concentrations in both raw and treated wastewater, suggesting their persistence throughout the treatment stages [62]. The global contamination of ATVs in WWTPs is documented in Table 2, providing comprehensive information on their presence and levels. Prasse et al. conducted a comprehensive study in Germany, unveiling the presence of various ATVs such as acyclovir, abacavir, lamivudine, nevirapine, oseltamivir, penciclovir, ribavirin, stavudine, zidovudine, and oseltamivir carboxylate in influent and effluent streams of WWTPs, as well as in the surface water of the Ruhr River [62]. Their investigation further revealed the presence of antiviral drug contamination in river waters, with concentrations ranging from lower ng/L levels to a maximum of 190 ng/L for acyclovir and 170 ng/L for zidovudine. ATVs were detected in both raw and treated water samples from various countries, including Germany [63,64], South Africa [65,66,67], and China [16], indicating a global occurrence of contamination. The presence of ATVs in diverse water matrices underscores the urgent need for comprehensive studies and the innovative design of advanced treatment strategies to effectively remove or eliminate these contaminants.

## 4. Photocatalytic Degradation of ATVs

ATVs, a category of emerging contaminants, play a critical role in combating a wide spectrum of viral infections, encompassing HIV, hepatitis, influenza A and B, herpes, Ebola, and a plethora of other viral pathogens [68]. ATVs can potentially enter the environment through various sources, including effluents from WWTPs, hospital waste streams, and pharmaceutical industrial discharges [61]. Scientific documentation reveals that prevailing treatment technologies employed in conventional WWTPs exhibit limited efficacy in eliminating ATVs from wastewater streams. Thus, it is imperative to investigate and develop advanced source treatment methods in order to effectively mitigate the presence of ATVs in environmental water sources. Heterogeneous photocatalysis emerges as a highly promising approach, offering an excellent opportunity for the efficient elimination of ATVs and other emerging contaminants through the synergistic interplay between catalyst materials and light irradiation. Heterogeneous photocatalysis holds notable advantages, prominently encompassing the absence of necessitating supplementary chemicals, low energy demand, operation under mild conditions, and overall cost effectiveness [69,70,71]. Harnessing the capabilities of photocatalysis, numerous photocatalytic materials have been meticulously developed, demonstrating substantial potential in manifesting robust photocatalytic activity.

### 4.1. Principle of Photocatalytic Degradation

The progression of events typically observed in heterogeneous photocatalysis commences with the excitation of the catalytic materials. Upon irradiation, the activation process involves the promotion of electrons (e^−^) from the valence band (VB) to the conduction band (CB), resulting in the formation of e^−^–hole (h^+^) pairs, as shown in Equation (1) [23]. The e^−^–h^+^ pairs exhibit an exceedingly brief lifespan, typically on the order of a few nanoseconds, necessitating their prompt separation to prevent recombination. The separation of the e^−^–h^+^ pairs can be achieved through the presence of e^−^ donors and acceptors, facilitating the migration of e^−^ and h^+^ to the surface of the catalyst, where they actively engage in redox reactions. In a majority of instances, the h^+^ readily engage with water molecules, giving rise to the generation of •OH (Equation (2)), while e^−^, if oxygen is present, can be captured to yield superoxide radicals (•O_2_^−^) (Equation (3)). Subsequently, these radicals effectively initiate the decomposition of the organic pollutants (OPs) through reactive interactions, as illustrated in Equation (4), or alternatively, they may propagate a cascade of reactions, generating an increased abundance of radicals (Equations (5) and (6)). In certain instances, the adsorbed pollutant molecules can undergo direct reduction facilitated by the presence of CB e^−^ (Equation (7)). In addition to the reactions described by Equations (2) and (3), the degradation of organic pollutants via photocatalysis can involve the generation of radical species through indirect pathways, as shown in the subsequent Equations (8)–(10).

(1) $$\mathrm{Catalyst}\;\overset{hv}{\to }e_{CB}^{-}\;+h_{VB}^{+}$$

(2) $$h_{VB}^{+}+H_{2}O\;\to •\mathrm{OH}+H^{+}$$

(3) $$e_{CB}^{-}+O_{2}\to •{O_{2}}^{-}$$

•OH + OPs → OPs_ox_ (4)

2•O_2_^−^ + 2H_2_O → H_2_O_2_ + O_2_ + 2OH^−^ (5)

H_2_O_2_ → 2•OH (6)

(7) $$e_{CB}^{-}+\mathrm{OPs}\to {\mathrm{OPs}}_{\mathrm{red}}$$

•O_2_^−^+ H^+^ → •HO_2_ (8)

2•HO_2_ → H_2_O_2_ + O_2_ (9)

H_2_O_2_ → 2•OH (10)

### 4.2. Semiconductor-Based Photocatalytic Degradation of ATVs

#### 4.2.1. Metal Oxide Semiconductors

**Figure 1:**
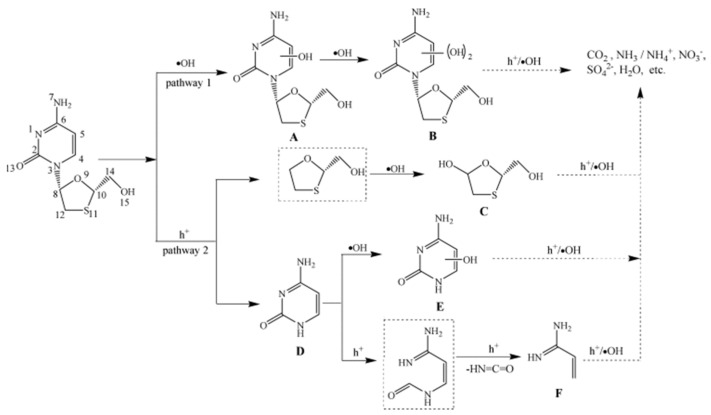
Proposed photocatalytic degradation mechanism of lamivudine in TiO_2_ suspension. Copyright Year 2011, *Journal of Hazardous Materials* © Elsevier Pvt Ltd.

Among the vast array of metal oxide photocatalysts, TiO_2_ emerges as a highly favored candidate, renowned not only for its efficacy in degrading organic pollutants but also for its potential in addressing the challenge of contamination related to ATVs [72,73]. In the field of photocatalytic decomposition of ATVs, extensive research in the literature indicates that all conducted studies have consistently utilized P25 TiO_2_ obtained from diverse suppliers, along with visible range irradiation as the predominant experimental approach [74,75]. Remarkable degradation efficiencies exceeding 95% were consistently achieved across all experimental cases employing P25 TiO_2_, underscoring the efficacy of this photocatalyst in the degradation process. Nevertheless, the literature revealed significant heterogeneity in the observed mineralization efficiencies during the photocatalytic degradation of ATVs. For instance, the mineralization of acyclovir [76] and oseltamivir [77], in contrast to the nearly complete degradation of the parent compounds, exhibited minimal to negligible levels (<10%). These findings indicated the inherent resistance of the intermediates to photocatalytic decomposition, as demonstrated in the reported studies. In another study, An et al. reported a mineralization efficiency of approximately 20% alongside the complete degradation of lamivudine within a duration of 1 h, under the specified experimental conditions [75]. The optimization of conditions involved setting the TiO_2_ concentration to 1.00 g/L, maintaining a pH value of 6.7, and utilizing an initial lamivudine concentration of 60 µM. The escalation in TiO_2_ concentration leads to an increased excitation of TiO_2_ particles by UV light, consequently yielding higher amounts of reactive species and subsequently higher rate constants. Nevertheless, as the TiO_2_ concentrations are further increased from 1.00 g/L to 3.00 g/L, there is a rapid decline in light penetration, leading to reduced excitation and deactivation of TiO_2_ particles, likely due to TiO_2_ particle–particle collisions. The plausible photocatalytic degradation mechanism of lamivudine in TiO_2_ suspension is shown in Figure 1. In the case of oseltamivir, although more than 95% of the compound was degraded within the initial 50 min of the experiment, after 6 h of irradiation, 46% to 57% of the total organic carbon (TOC) still persisted in the solution, suggesting the presence of numerous intermediate species during the photocatalytic process. The ATVs, including 1-amantadine, 2-amantadine, rimantadine, and acyclovir, exhibited high degrees of mineralization (>80%), indicating their susceptibility to degradation and mineralization through photocatalysis [74,78]. In the presence of AEROIXE TiO_2_ P25, zanamivir underwent complete degradation within 1 min [79]. The dependence on the amount of TiO_2_ was investigated. The findings clearly demonstrate that an increase in the initial amount of TiO_2_ leads to a correspondingly higher transformation rate. Subsequently, increasing the TiO_2_ amount to 10 mg does not lead to further acceleration of zanamivir degradation. An increase in the TiO_2_ amount results in elevated suspension turbidity, leading to subsequent scattering effects. On the other hand, its primary degradation product, guanidine, displayed remarkable resistance to degradation under the same experimental conditions. The response of ATVs to photocatalytic treatment is highly dependent on the specific experimental conditions employed. For example, the light-activated PMS demonstrated the capability to reduce the concentration of maraviroc by half within 7 min of irradiation [80]. However, when combined with TiO_2_, the half-life was reduced to 0.47 min, a remarkable decrease of over 67,000 times compared to direct photolysis. Therefore, direct comparisons between studies are currently challenging due to the lack of similarities among the investigations conducted. A summary of the photocatalytic degradation of different ATVs using doped metal oxides can be found in Table 3.

#### 4.2.2. Doped Metal Oxide Semiconductors

Doping and sensitization techniques offer the potential for shifting the light absorption response of semiconductors towards the visible light range [81]. Additionally, this process prolongs the lifetime of e^−^ and h^+^ within the semiconductor materials. The metal ion doping or co-doping of metals and non-metals, along with metal oxide modification through the use of capping agents, represent highly promising approaches to mitigate charge carrier recombination [82]. For instance, Pazoki et al. reported that a TiO_2_/Ag photocatalyst was investigated for its effectiveness in degrading and removing dexamethasone from aqueous matrices under both visible and UV light irradiation [83]. Under the optimal dosage of operational parameters, the maximum degradation efficiency of 82.3% was achieved under UV irradiation, while a degradation efficiency of 71.5% was attained under visible-light irradiation. Similarly, (Ag,Cu) co-doped TiO_2_ photocatalysts were prepared using the sol–gel method, and the removal efficiency of acyclovir reached 98%, which is 2.34 times higher than TiO_2_ [84].

#### 4.2.3. Heterojunction Semiconductors

**Figure 2:**
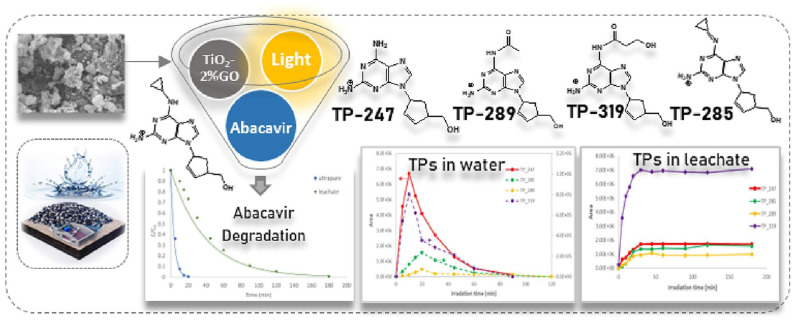
Schematic of the intermediate transformation products during the photocatalytic degradation of abacavir. Copyright Year 2023, *Journal of Photochemistry & Photobiology, A: Chemistry* © Elsevier Pvt Ltd.

**Figure 3:**
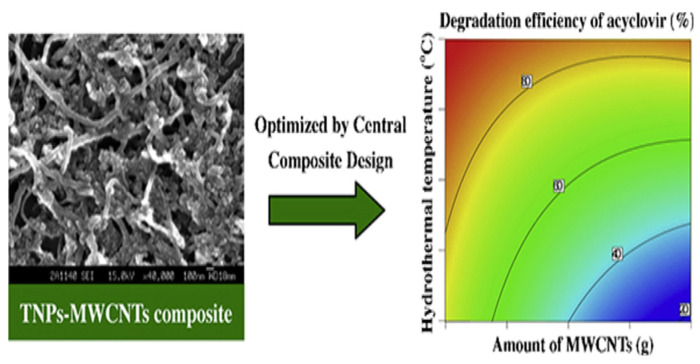
The relationship between the amount of MWCNTs and the degradation efficiency of acyclovir. Copyright Year 2014, *Applied Catalysis A: General* © Elsevier Pvt Ltd.

Heterojunction semiconductors have emerged as a promising strategy in the quest for efficient photocatalytic systems, particularly in harnessing the potential of visible light [85,86,87]. Graphene oxide (GO) holds great promise in the field of photocatalysis owing to its unique characteristics, including its two-dimensional geometry, expansive surface area, and excellent conductivity, which enable it to effectively engage all three mechanisms of photocatalytic enhancement, namely (i) heightened adsorptivity towards pollutants, (ii) facile separation of charge carriers, and (iii) an extended range of light absorption [88,89,90]. Considering the aforementioned factors, Evgenidou et al. synthesized GO-TiO_2_ nanocomposites and evaluated their effectiveness in degrading abacavir [91]. They demonstrated remarkable photocatalytic efficiency in degrading abacavir. Significantly, the composite containing 2% GO content exhibited superior degradation rates, completely eliminating the target compound within a mere 20 min of treatment. Subsequently, an investigation was conducted into the photocatalytic reaction mechanism, along with the identification of transformation products generated during the reaction process (Figure 2). In addition, a composite photocatalyst consisting of TiO_2_ nanoparticles and multi-walled carbon nanotubes (TNPs–MWCNTs) was synthesized using a straightforward soft-template hydrothermal method, and its composition was optimized using a center-composite design (CCD) approach [92]. The effects of these components on the photocatalytic activity of the resulting composites towards acyclovir degradation in water were investigated. Based on the combined theoretical and experimental findings (Figure 3), the TNPs–MWCNTs composite photocatalyst synthesized under optimized conditions, including a hydrothermal temperature of 240 °C, 0.06 g of MWCNTs, 1.10 g of TBT, and 0.10 g of Pluronic P123, demonstrated the highest photocatalytic degradation efficiency for acyclovir, reaching up to 98.6%.

**Figure 4:**
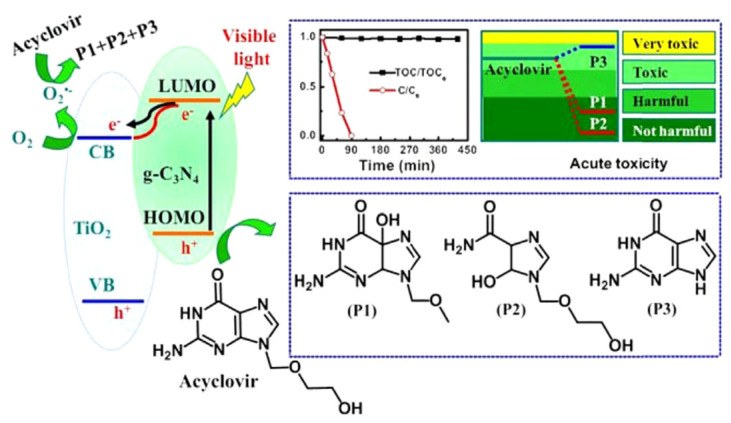
Schematic of the photocatalytic degradation via acyclovir by g-C_3_N_4_/TiO_2_ hybrid photocatalysts. Copyright Year 2016, *Applied Catalysis B: Environmental* © Elsevier Pvt Ltd.

**Figure 5:**
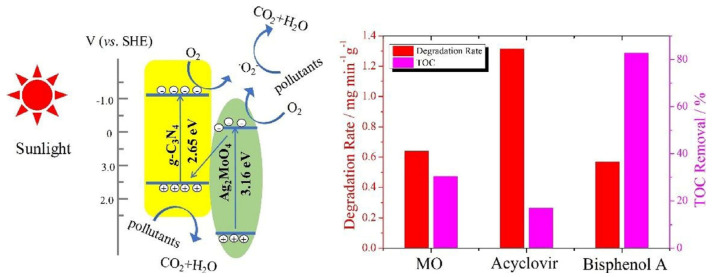
Photocatalytic degradation mechanism over Ag_2_MoO_4_/g-C_3_N_4_ under sunlight irradiation. Copyright Year 2018, *Catalysis Today* © Elsevier Pvt Ltd.

**Figure 6:**
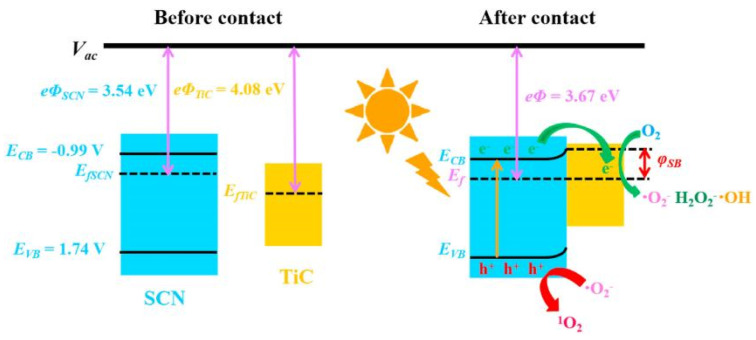
TiC/SCN photocatalytic mechanism. Copyright Year 2022, *Chemosphere* ©Elsevier Pvt Ltd.

Graphitic carbon nitride (g-C_3_N_4_) has gained considerable research interest for its potential in degrading organic pollutants. This attraction arises from its low cost, appropriate electronic structure, and high chemical stability, making it a promising materials in the field [93,94]. Li et al. employed TiO_2_, g-C_3_N_4_, and a hybrid of g-C_3_N_4_ and TiO_2_ (g-C_3_N_4_/TiO_2_) for the degradation of acyclovir [76]. As a result, the degradation of acyclovir under TiO_2_ photocatalysis exhibited minimal advancement even after 5 h of irradiation. However, the incorporation of g-C_3_N_4_ significantly enhanced the degradation efficiency. Notably, the implementation of the g-C_3_N_4_/TiO_2_ hybrid as a photocatalyst achieved the complete degradation of acyclovir within a remarkable 4 h. As shown in Figure 4, it is evident that the hybrid catalyst displayed a significantly reduced bandgap, facilitating efficient charge carrier separation. Furthermore, Ag_2_MoO_4_ nanoparticles encapsulated in g-C_3_N_4_ (Ag_2_MoO_4_/g-C_3_N_4_) were synthesized with a facile in-situ precipitation method [95]. The band structure of Ag_2_MoO_4_ facilitated a synergistic effect with g-C_3_N_4_, leading to enhanced solar light absorption and a reduced recombination rate of photo-induced e^−^–h^+^ pairs. Therefore, under sunlight irradiation, the Ag_2_MoO_4_/g-C_3_N_4_ samples demonstrated markedly superior photocatalytic activity in the degradation of acyclovir, surpassing the performance of pristine g-C_3_N_4_ (Figure 5). In order to remove arbidol hydrochloride (ABLH), a novel photocatalyst composed of Ti_3_C_2_ MXene and supramolecular g-C_3_N_4_ (TiC/SCN) was prepared via a self-assembly method [96]. The 0.5TiC/SCN photocatalyst achieved an impressive ABLH removal efficiency of 99% within 150 min under visible-light illumination. Moreover, in the presence of real sunlight illumination, the 0.5TiC/SCN photocatalyst demonstrated a remarkable ABLH removal efficiency of 99.2% within a shorter duration of 120 min, surpassing the performance of the commercial P25 TiO_2_. The elucidation of the potential mechanism associated with the TiC/SCN Schottky junction is presented in Figure 6. The calculated CB potential of SCN was determined to be −0.99 V versus NHE, exhibiting a higher negative value compared to the redox potential of O_2_/•O_2_^−^ (−0.33 V versus NHE). This suggested the feasibility of O_2_ reduction to generate •O_2_^−^ and H_2_O_2_. The determined VB potential of SCN was found to be more negative than the redox potentials of OH^−^/•OH (1.99 V versus NHE) and H_2_O/•OH (2.37 V versus NHE), suggesting that the direct generation of •OH was not feasible. Consequently, the establishment of a space charge layer occurred on the SCN side, leading to the upward curvature of the energy band and the creation of a Schottky barrier [97]. The generation of reactive oxygen species (ROS) was facilitated, thereby enhancing the photocatalytic performance of 0.5TiC/SCN. Subsequently, following four consecutive cycles, the removal efficiency of ABLH by 0.5TiC/SCN decreased from 99.1% to 96.3% within 150 min. These findings provide additional evidence of the stability via 0.5TiC/SCN, suggesting its suitability for practical applications. In addition, a novel nanocomposite, CuSm_0.06_Fe_1.94_O_4_@g-C_3_N_4_, exhibiting exceptional magnetic, electrochemical, and optical properties, was successfully synthesized through a hydrothermal method. Significant removal efficiencies were achieved in the photodegradation of various dyes, including congo red, tartrazine, and metanil yellow, as well as pharmaceutical compounds such as carbamazepine, zidovudine, and acetaminophen [98]. About 71.5% of zidovudine was removed in 140 min.

**Figure 7:**
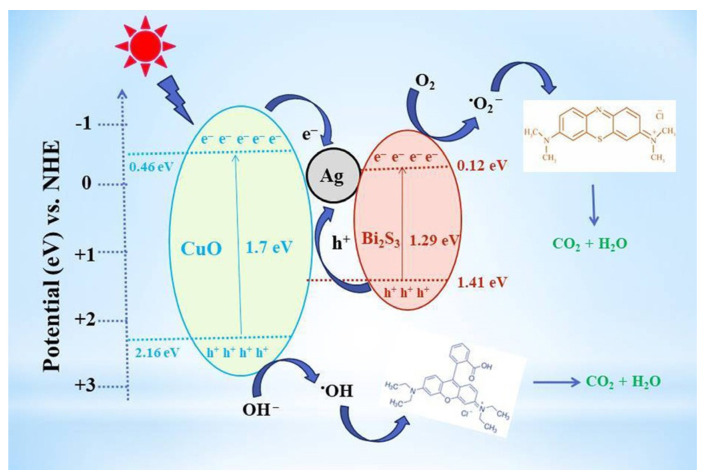
CuO@Ag@Bi_2_S_3_ photocatalytic mechanism of stavudine and zidovudine. Copyright Year 2022, *New Journal of Chemistry* © Royal Society of Chemistry Ltd.

**Figure 8:**
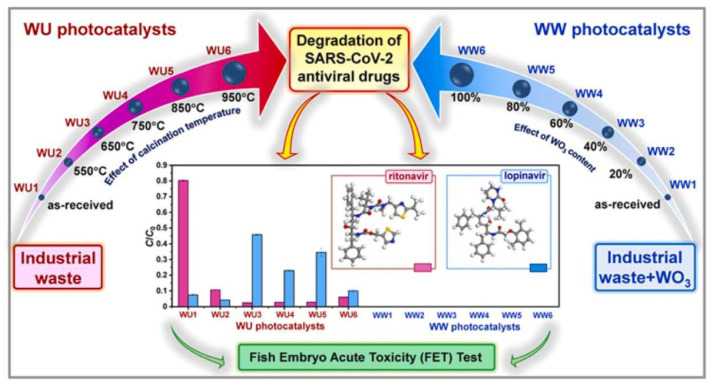
Photodegradation of ritonavir and lopinavir by the synthesized WU and WW photocatalysts. Copyright Year 2022, *Journal of Hazardous Materials* © Elsevier Pvt Ltd.

**Figure 9:**
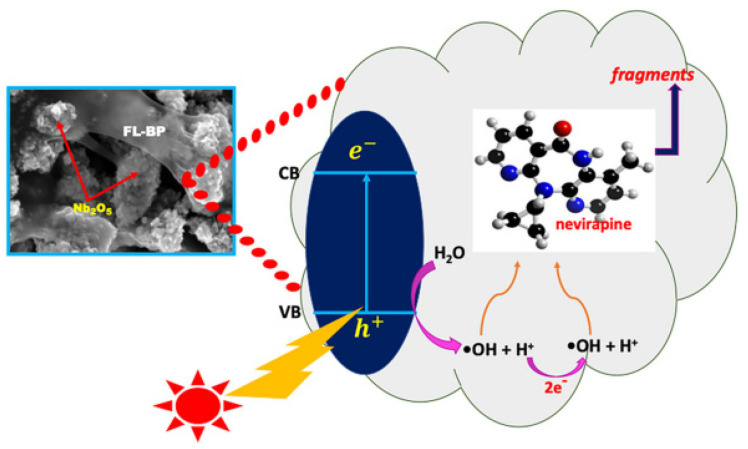
Possible photodegradation mechanism of nevirapine by the synthesized FL-BP@Nb_2_O_5_ photocatalysts. Copyright Year 2020, *Chemosphere* © Elsevier Pvt Ltd.

Hu et al. successfully synthesized a novel nanoscale photocatalyst, Bi_4_VO_8_Cl, using a hydrothermal synthesis method [99]. The synthesized material was thoroughly characterized to gain insights into its structural and functional properties. The catalytic performance of this photocatalyst was evaluated by investigating its effectiveness in the degradation of six pharmaceutical compounds, namely metronidazole, aciclovir, levofloxacin hydrochloride, sulfonamide, adrenaline hydrochloride, and ribavirin, in aqueous solutions under visible-light irradiation. Among them, aciclovir achieved complete mineralization within 10 h under visible-light irradiation. Ayodhya et al. reported the synthesis of a novel Z-scheme catalyst, a ternary composite of CuO@Ag@Bi_2_S_3_, by homogeneously precipitating Ag particles onto CuO and Bi_2_S_3_ using an ultrasonication method [100]. The CuO nanoparticles were fabricated through the reduction of a Cu(II)-Schiff base complex. The remarkable catalytic activity of the CuO@Ag@Bi_2_S_3_ ternary composite in the degradation of HIV drugs, such as stavudine and zidovudine, is clearly demonstrated in Figure 7. Upon the incorporation of Ag NPs into the CuO@Bi_2_S_3_ composite, a notable increase in intensity was observed, accompanied by the broadening of the absorption band in the visible region. For stavudine, the CuO@Ag@Bi_2_S_3_ composite achieved a remarkable maximum removal efficiency of approximately 92.14% within a reaction time of 30 min. In the case of zidovudine, the maximum removal efficiency was found to be 87.42%. The CuO@Ag@Bi_2_S_3_ exhibited a significantly higher removal efficiency compared to CuO, Bi_2_S_3_, Ag@Bi_2_S_3_, Ag@CuO, and CuO@Bi_2_S_3_ in both scenarios. This notable enhancement could be attributed to the relatively-low-molar absorption coefficients of the drugs and the exceptional adsorption capacity of the composite in aqueous media [101]. The investigation revealed that •O_2_^−^ and h^+^ emerged as the prevailing active species during the photocatalytic process. Additionally, the X-ray diffraction (XRD) patterns from the first and fifth cycles of the prepared CuO@Ag@Bi_2_S_3_ ternary composite displayed a consistent structure and intensity, providing robust evidence for its favorable stability. In a subsequent study, the synthesis of cost-effective multiphase photocatalysts via a straightforward calcination process utilizing industrial waste obtained from ammonium molybdate production (referred to as WU photocatalysts) combined with WO_3_ (referred to as WW photocatalysts) was reported by Hojamberdiev et al. [102]. The multiphase photocatalysts demonstrated a remarkable efficiency of 95% in the photocatalytic degradation of ritonavir under 15 min of visible-light irradiation. In contrast, a longer irradiation time of 60 min was required to achieve a 95% efficiency in the photocatalytic degradation of lopinavir. According to the results of the recyclability test conducted using WU6, the synthesized multiphase photocatalyst exhibited a slight decrease in efficiency after the third cycle, underscoring its notable stability. Moreover, the investigation on the ecotoxicity of photocatalytically treated ritonavir-containing wastewater using zebrafish (*Danio rerio*) embryos revealed no signs of toxicity. However, a contrasting trend was observed for photocatalytically treated lopinavir-containing wastewater, indicating potential adverse effects. During the photocatalytic removal of lopinavir, the intermediates or by-products generated exhibited certain levels of toxicity (Figure 8). In another study, Bhembe et al. successfully synthesized a FL-BP@Nb_2_O_5_ photocatalyst and evaluated its performance in the photodegradation of nevirapine, comparing its degradation efficiency with that of pristine Nb_2_O_5_ [103]. The FL-BP@Nb_2_O_5_ exhibited an augmented light-harvesting capacity owing to the reduction in bandgap, attributable to the synergetic effects occurring at the BP and Nb_2_O_5_ interface. The degradation parameters were systematically optimized, revealing that the most optimized conditions to achieve the highest degradation efficiency for nevirapine were found when using its lowest concentration of 5 ppm, with a catalyst loading of 15 mg at a working pH of 3 for 3%FL-BP@Nb_2_O_5_. Subsequently, the p-n junction formed in the composite material (absent in pristine Nb_2_O_5_) was elucidated to facilitate the cross-flow of e^−^ and h^+^, promoting e^−^ migration to the surface of the photocatalyst and their active participation in the degradation process (Figure 9). The performances of different heterojunction semiconductors for ATV degradation are summarized in Table 4. Typically, single-component, semiconductor-based photocatalysts demonstrate a limited light absorption range and relatively low redox ability. Hence, considerable research efforts have been directed towards the exploration of modified semiconductor-based compounds, particularly through heterojunction construction, and they have exhibited superior performance in the photocatalytic degradation of ATVs. In summary, high ATV photocatalytic degradation efficiency is demonstrated by the heterojunction semiconductors, owing to their large specific surface area, enhanced visible light absorption, and accelerated interfacial charge transfer and separation.

## 5. Challenges and Future Perspectives

During each epidemic, pandemic, or outbreak, a substantial volume of medications are administered to control the disease among the affected or susceptible population. As a result, a significant proportion of these drugs, either in their parent form or as metabolites, find their way into the aquatic environment. ATVs have been extensively detected in various water matrices, such as influents and effluents of WWTPs, groundwater, surface water, and even drinking water, as evidenced by the available scientific literature. The observed concentrations range from ng/L to mg/L, indicating the limited effectiveness of conventional or advanced treatment methods in adequately removing these compounds from wastewater and ensuring the quality of drinking water. The collective efforts of the scientific community are required to establish a comprehensive database in this field, encompassing the occurrence and fate of ATVs in environmental water sources. The current COVID-19 pandemic serves as a stark reminder of the pressing need to address the inadequacy in treating wastewater and preventing the dispersion of contaminants in diverse environmental matrices, thereby mitigating potential adverse impacts.

Semiconductor-based photocatalysis holds significant promise as an environmentally sustainable approach for the effective removal of pollutants from both water and air, garnering considerable attention in the field of green chemistry. Nevertheless, the widespread implementation and commercialization of this technique face notable challenges, including the limited efficiency of photocatalysts under natural light conditions, the need for catalyst reusability, the optimization of operating conditions, and the development of suitable reactor designs [104,105,106]. Prior to establishing on large-scale implementation, it is imperative to amass a comprehensive body of research data elucidating the behavior of ATVs in photocatalytic treatment systems, with the ultimate aim of attaining complete degradation and mineralization efficiencies. A comprehensive and rigorous investigation of degradation kinetics, mechanisms, treatment parameters, and interaction dynamics is essential to enhance the efficacy of treatment systems, enabling the achievement of superior levels of efficiency and performance. More research is needed on the photocatalytic degradation of ATVs. Researchers have shown particular interest in oseltamivir, followed by acyclovir, lamivudine, zidovudine, and amantadine among the wide range of available ATVs. Photocatalysis has demonstrated high efficiency in the degradation of hydrophilic compounds like ATVs, which are likely to selectively adsorb onto the relatively polar catalytic surfaces. Given the susceptibility of ATVs to degradation by •OH, heterogeneous photocatalysis within AOPs emerges as a highly favorable option. A comprehensive research effort is necessary to investigate the varied responses of different ATVs, as their behavior and degradation pathways may exhibit significant variations, necessitating individualized studies for each drug. Secondly, it is worth noting that the majority of existing investigations have primarily concentrated on the degradation of the parent compound, yielding impressive results in terms of achieving notable degradation efficiencies. Nevertheless, it is crucial to highlight that these same reports caution about the potential persistence of photocatalytic degradation intermediates or byproducts within the system, which may exhibit equal or even higher toxicity compared to the parent compound. This serves as a reminder that achieving complete mineralization should be the primary objective in the degradation of ATVs. Thirdly, there is a dearth of research examining the photocatalytic degradation of ATVs in actual wastewater systems, as evidenced by the limited literature available in this area.

## 6. Conclusions

The progress in the field of ATVs has been primarily driven by the need to effectively combat viral infections and mitigate their impact on human health. Nevertheless, the presence of ATVs as emerging contaminants in the environment has garnered significant attention. Therefore, there is a pressing need to devise a highly efficient method for the complete elimination of ATVs. The majority of this review is focused on the categorization, occurrence and semiconductor-based photocatalytic degradation of ATVs. Semiconductor-based photocatalysis presents a promising option for the degradation of ATVs. A wide range of photocatalytic materials have been developed, demonstrating significant potential for photocatalytic activity. Extensive investigation is required to optimize the treatment system considering the significant impact of operational conditions on photocatalytic treatment. Semiconductor-based photocatalysis utilizing TiO_2_ for the degradation of ATVs has demonstrated cost-effectiveness, taking into account energy requirements and the overall process efficiency. The construction of heterojunction semiconductor systems exhibits intriguing prospects due to their synergistic effects and potential for enhanced performance. Further investigations are recommended to expand the existing knowledge on the photocatalytic degradation of ATVs and contribute to the growing body of research in this field. Overall, additional research is necessary to develop effective treatment design strategies and scale them up for practical implementation at operational levels.

## Figures and Tables

**Table 1:** Main types of ATVs.

Virus	ATVs	CAS Number	Formula	Chemical Structure	Molecular Weight (MW) (g/mol)
HIV	abacavir	136470-78-5	C_14_H_18_N_6_O	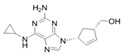	286.33
	bictegravir sodium	1807988-02-8	C_21_H_17_F_3_N_3_NaO_5_	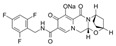	471.36
	lamivudine	131086-21-0	C_8_H_11_N_3_O_3_S	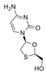	229.26
	nevirapine	129618-40-2	C_15_H_12_N_2_O_4_	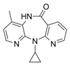	266.29
	stavudine	3056-17-5	C_10_H_12_N_2_O_4_	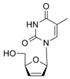	224.21
	zidovudine	30516-87-1	C_10_H_13_N_5_O_4_	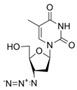	267.24
HSVs	acyclovir	59277-89-3	C_8_H_11_N_5_O_3_	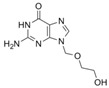	225.20
	famciclovir	104227-87-4	C_14_H_19_N_5_O_4_	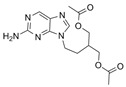	321.33
	penciclovir	39809-25-1	C_10_H_15_N_5_O_3_	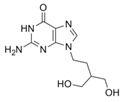	253.26
Influenza	amantadine	768-94-5	C_10_H_17_N	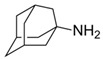	151.24
	oseltamivir	196618-13-0	C_16_H_28_N_2_O_4_	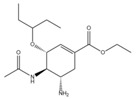	312.40
	zanamivir	139110-80-8	C_12_H_20_N_4_O_7_	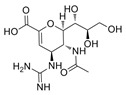	332.31
SARS-CoV-2	favipiravir	259793-96-9	C_5_H_4_FN_3_O_2_	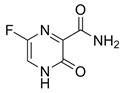	157.10
	remdesivir	39809-25-1	C_27_H_35_N_6_O_8_P	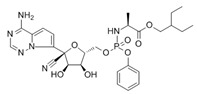	602.57

**Table 2:** Occurrence of ATVs in WWTPs influents and effluents worldwide.

ATV	Concentration ng/L (Min–Max)		Country	References
	Influent	Effluent		
acyclovir	1780–1990	27–53	Germany	[62]
lamivudine	210–720	ND		
nevirapine	4.8–21.8	7–32		
oseltamivir	0–11.9	9–16		
zidovudine	310–380	98–564		
stavudine	11.6–22.8	ND		
acyclovir	ND	ND		[63]
emtricitabine	ND	130		
emtricitabine carboxylate	ND	120–1000		
abacavir	60–140	ND		[64]
abacavir carboxylate	180–500	100–280		
emtricitabine	100–980	59–170		
emtricitabine carboxylate	24–25	140–480		
acyclovir	520–4980	0–270		
abacavir	0–14,000	ND	South Africa	[65]
zidovudine	6900–53,000	87–500		
nevirapine	670–2800	540–1900		
lamivudine	840–2200	0–130		
efavirenz	24,000–34,000	20,000–34,000		
acyclovir	0–406	0–205	China	[16]
ribavirin	ND	ND		
zidovudine	ND	ND		

ND, not detected.

**Table 3:** Metal oxide semiconductors photocatalytic degradation of ATVs reported in the literature.

ATV	Initial Concentration (μM)	Catalyst	Catalyst Dose (mg/L)	UV Range (nm)	Removal (%)	Rate Constant (min^−1^)	References
oseltamivir	24	P25	20	365	96	0.040	[78]
acyclovir	50	P25	500	365	100	–	[75]
lamivudine	100	P25	1000	365	>95	0.0542	[76]
1–amantadine	100	P25	1000	365	100	0.076	[79]
2–amantadine	100	P25	1000	365	100	0.084	[79]
rimantadine	100	P25	1000	365	100	0.102	[79]
zanamivir	0.3	AEROIXE TiO_2_ P25	17.7	380–420	100	–	[80]

**Table 4:** Heterojunction semiconductors photocatalytic degradation of ATVs reported in the literatures.

ATVs	Initial Concentration (μM)	Catalyst	Catalyst Dose (mg/L)	UV Range (nm)	Removal (%)	Rate Constant (min^−1^)	References
abacavir	10	GO-TiO_2_	100	solar spectrum	99.4	0.2610	[91]
acyclovir	10	TNPs-MWCNTs	400	365	98.6	-	[92]
acyclovir	10	g-CN/TiO_2_	300	>420	100	0.0076	[76]
acyclovir	10	Ag_2_MoO_4_/g-C_3_N_4_	250	>420	100	-	[95]
arbidol hydrochloride	10	Ti_3_C_2_ MXene/g-C_3_N_4_	100	>420	99.2	0.0295	[96]
zidovudine	10	CuSm_0.06_Fe_1.94_O_4_@g-C_3_N_4_	1200	>420	71.5	0.0081	[98]
acyclovir	10	Bi_4_VO_8_Cl	50	200–780	100	-	[99]
ribavirin	10	Bi_4_VO_8_Cl	50	200–780	100	-	[99]
stavudine	10	CuO@Ag@Bi_2_S_3_	20	365	92.1	-	[100]
zidovudine	10	CuO@Ag@Bi_2_S_3_	20	365	87.4	-	[100]
lopinavir	10	ammonium molybdate (WU and WWphotocatalysts)	400	500–550	95	-	[102]
ritonavir	10	ammonium molybdate (WU and WWphotocatalysts)	400	500–550	95	-	[102]
nevirapine	5	FL-BP@Nb_2_O_5_	100	>420	68	0.0152	[103]

## Data Availability

Data will be granted upon request.
